# Experience feedback committees: A way of implementing a root cause analysis practice in hospital medical departments

**DOI:** 10.1371/journal.pone.0201067

**Published:** 2018-07-26

**Authors:** Patrice François, André Lecoanet, Alban Caporossi, Anne-Marie Dols, Arnaud Seigneurin, Bastien Boussat

**Affiliations:** 1 Public Health Department, Grenoble-Alpes University Hospital, Grenoble, France; 2 Laboratory TIMC, UMR 5525, CNRS, Grenoble-Alpes University, Grenoble, France; Medical University Graz, AUSTRIA

## Abstract

**Background:**

The experience feedback committee (EFC) is a tool designed to involve medical teams in patient safety management, through root cause analysis (RCA) within the team.

**Objectives:**

To investigate the functioning of EFCs in the departments of a large university-affiliated hospital in France and to consider its potential contribution to the management of patient safety.

**Methods:**

Cross-sectional, observational study, based on an analysis of the documents produced by the EFCs for 1 year. Data were collected independently by two investigators in meeting minutes, adverse event reports and event analysis reports.

**Results:**

The study included all 20 EFCs operating in the hospital's medical departments. During the study year, committees held 164 meetings, reviewed 1707 adverse events, conducted 91 event analyses and decided on 206 corrective actions. The median number of corrective actions adopted by each EFC was five actions (range, 0–62). A root cause analysis (RCA) was present in 76% of the analysis reports, and these analyses were complete in only 23% of the reports. There was also a lack of planning corrective actions: an implementation deadline was only defined in 26% of the actions.

**Conclusions:**

Healthcare professionals adhered to the system-based approach to patient safety, but we observed difficulties in holding regular meetings and deviations from the theoretical framework. These findings confirm the difficulties of practicing RCA in the healthcare setting. Nevertheless, EFCs can be vectors of safety culture and teamwork.

## Introduction

The improvement of patient safety remains one of the greatest challenges of all healthcare systems.[1] Despite considerable initiatives over nearly 20 years aiming to improve safety in healthcare, progress remains slow and the results are modest.[2] The underlying reasons for this lack of progress include unquestionably complex factors related to the sociology of healthcare organizations. [3] It is now recognized that a concrete way to reduce adverse events is the development of a patient safety culture shared among hospital care providers.[3] Accordingly, a wide variety of interventions has been developed to directly involve healthcare professionals in safety management, such as the Comprehensive Unit-based Safety Program (CUSP) and the TeamSTEPPS in the United States. [4–7]

In France, the vast majority of healthcare facilities have a similar program called the experience feedback committee (EFC). [8] The main principle of this management system is to involve the medical team in root cause analysis (RCA). The EFC members usually meet monthly to examine reported incidents that occurred in their department. They choose priority incidents that need to be analysed and propose corrective actions. A recent study reported a favourable association between involvement in EFC activities and the patient safety culture among hospital care providers.[9] Moreover, several studies have reported promising adherence of medical teams involved in EFC in emergency, psychiatry and pharmacy departments.[10–12] However, these studies identified several limitations related to the initial EFC framework. Despite its wide implementation in the French healthcare system, the functioning of the EFC program has never been evaluated at the level of an entire hospital. This lack of scientific evaluation constitutes a barrier to improving EFCs. Indeed, better knowledge of the strengths and limitations of EFC functioning is needed to adjust its initial framework and make it more efficient and better manage patient safety.

Through the analysis of the 20 EFCs implemented in a large university-affiliated hospital, the objective of this study was to investigate the functioning of the EFC and to consider its contribution to the management of patient safety.

## Methods

### Study design

We conducted a cross-sectional, observational study of EFCs established in the Grenoble University Hospital (France). The study was approved by the Institutional Research Ethics Committee of Southeastern France (Comité de Protection des Personnes,Sud Est V, France; IRB 6705).

### Setting

The study was conducted in a 1347-bed acute-care university hospital including 42 clinical and medical-technical departments.

The hospital’s central risk management system was described elsewhere.[12] Adverse and near-miss events are reported to a central safety unit using a voluntary internal reporting system based on a standardised reporting form. This unit is made up of one medical doctor, one pharmacist and one quality engineer. The events reported were classified by severity and risk area. The central safety unit directly investigates the most serious events and those involving several hospital departments. Other events are transmitted to the appropriate operator and the executives of the relevant departments. For departments where an EFC is implemented, the central safety unit addresses the reports of events to the EFC leader every month.

### Experience feedback committee framework

The functioning of the EFC is defined in local guidelines in accordance with the framework proposed by Air France Consulting.[8] The departments can obtain methodological assistance from the quality-assurance team.

The Committee is composed of volunteer representatives of the department’s various professions. A few days before the committee meeting, the EFC leader receives a file including the department’s events reports from the central safety unit. The Committee can also set up a specific reporting system for the EFC.

The committee meets regularly, usually once a month, according to a fixed schedule. Meetings last between 1 and 2 h. Committee meetings are conducted according to a standardised framework: 1) reading the list of reported events, 2) choosing a priority event to investigate, 3) choosing the professional responsible for the investigation, 4) reviewing the analysis carried out for the event chosen the previous month, 5) deciding on corrective actions and 6) monitoring on-going actions.

The investigation is carried out during the month following the EFC meeting by a designated person using the Orion method developed by aviation safety experts.[8] The main steps of the method are as follows: collecting data and existing guidelines describing the chronological facts that occurred before, during and after the event; describing the failures; looking for causes of errors and latent factors that could have contributed to the failures; setting up corrective actions; and writing a report of the analysis. Causes and latent factors are found in different areas such as political, organisational, working conditions, team functioning, procedures, actors and the patient.

### Study sample

All EFCs established in the hospital departments more than 1 year before were eligible. The purpose of the study was presented to the EFC leaders and their consent was was requested orally to participate in the study and to allow investigators to analyze the documents produced by the EFC. These documents did not contain any patient-level identifying information.

### Data collection

We used the data collection method previously described elsewhere.[12] All written documents produced by the EFC during a 1-year period before the inclusion were analysed. These documents included meeting minutes, event reports, event analysis reports, and all documents related to corrective actions decided by the EFC. Reported events were classified according to the source of the report, the type of event and the consequence for the patient, using the International Classification for Patient Safety.[13] Written reports from meetings were analysed using a standardised form that included the theoretical steps of an EFC meeting. The event analysis reports were analysed using a standardised form in accordance with the Orion method. The corrective actions were classified by type (i.e., organisation improvement, procedure writing, staff training, device improvement) and planning elements (i.e., designating a person in charge and setting a deadline for implementation). No direct or indirect identification of patients or healthcare professionals was possible in the data collected. The data were collected independently by two investigators (PF & BB). Differences in recording data were discussed until a consensus was reached.

### Statistical analysis

EFC baseline characteristics were reported as numbers and percentages for categorical variables, and median and interquartile range (IQR, 25^th^ and 75^th^ percentiles) for continuous variables. To identify the most potentially productive EFCs, we generated another variable based on the median number of corrective actions decided per year. Thus, EFCs that decided to implement a number of actions greater than the median were considered potentially more productive than the others. Secondarily, we compared the EFC characteristics across subgroups of EFCs defined by this dichotomised variable using the chi-square test or Fischer’s exact test, when appropriate, for categorical variables, and the Kruskal-Wallis test for continuous variables. *P*-values less than 0.05 were considered statistically significant. Analyses were performed using Stata 14.0 (Stata Corp, College Station, TX, USA).

## Results

From 2007 to 2014, EFCs were implemented in 20 of the 42 hospital departments, including seven medical departments (gastroenterology, infectious diseases, cardiology, pediatrics, internal medicine, vascular medicine, neurology), six medical-technical departments (nuclear medicine, pharmacy, sterilisation, biology, radiotherapy), five emergency or intensive care departments and two surgical departments. Five EFCs had periods from 6 to 26 months of inactivity resulting from staffing issues, such as resignation of the EFC leader or understaffing in the related department.

During the 1-year period studied, the EFCs held 164 meetings (2–12 per EFC). They examined 1707 reported events, conducted in-depth analysis of 91 events and decided on 206 corrective actions.

### EFC meetings and participants

Meeting minutes were found for 160 (98%) of the 164 committee meetings. These reports mentioned 351 participants including 99 physicians, 48 head nurses and 76 nurses or other paramedics (Table 1). The review of the month’s event reports was noted in almost all meeting minutes. The presentation of an event analysis report, a list of decided actions and the follow-up of previous actions were present in 58% of all meeting minutes.

**Table 1:** Information included in the meeting minutes of the 20 EFCs (*n* = 160 reports).

	All meetings		Per EFC	
Presence of information	*n*	%	Median	[IQR]
– Review of event reports	158	99	8	[7; 10]
– Choice of an event to investigate	98	61	5	[1; 8]
– Presentation of analysis report	93	58	5	[2; 7]
– List of actions decided	92	58	4	[2; 7]
– Follow-up of previous actions	93	58	5	[2; 8]
Meeting attendees	351	100	15.5	[14; 21]
– Physicians	99	28	4	[3; 6]
– Head nurses	48	14	2	[1; 4]
– Nurses and other paramedics	76	22	3	[1; 6]
– Students	51	15	1	[0;5]
– Others (secretary, technicians, etc.)	77	21	2	[0;6]

IQR = Interquartile range

### Events reported

Of the 20 EFCs, 11 used event reporting from the central unit for risk management, six used only the reports collected in the EFC department and three used the two sources of reports (Table 2). Most events occurred in the EFC department (83%) and were reported by the professionals of the department (86%). These events related mainly to medication issues (21%), organisation of care (20%) or medical devices (17%). Most of the reported events (91%) did not have harmful consequences for the patients. However, three patients died and 11 suffered severe harm.

**Table 2:** Characteristics of event reports reviewed by EFCs (*n* = 1707).

	All EFCs		Per EFC	
	*n*	%	Median	[IQR]
Reporting route				
Central unit of risk management	1195	67	34	[0; 84]
EFC department	585	33	0	[0; 41]
Individual who reports				
EFC department staff	1475	86	50	[31; 106]
Other staff	232	14	3	[0; 11]
Location of occurrence				
EFC department	1422	83	49	[31; 103]
Other department	285	17	13	[2; 18]
Topics of event				
Medications	365	21	3	[0; 15]
Care organisation	337	20	10	[5; 25]
Medical device and equipment	298	17	8	[1; 10]
Care process and practices	253	15	3	[1; 7]
Patients and relatives	126	7	1	[0; 4]
Environment: premises, hygiene	125	7	3	[0; 6]
Staff	96	5	1	[0; 1]
Patient records	43	3	1	[0; 2]
Other^†^	64	5	1	[0; 2]
Severity of reported events				
Event without harm	1555	91	44	[24; 99]
Minor harm	96	6	3	[1; 7]
Moderated harm	42	2	1	[0; 2]
Severe harm	11	1	0	[0; 0]
Death	3	0	0	[0; 0]

Abbreviation: IQR, interquartile range (i.e., 25^th^ and 75^th^ percentiles)

† Transfusion, nosocomial infections, food, medical gases, bed availability, etc.

### Root cause analysis of events

Among all the events reviewed, 98 were selected for in-depth analysis. An analysis report presented at a committee meeting was produced for 91 event investigations, and 72 of them (79%) were presented according to the format of the Orion method (Table 3). The data collection procedures were specified by only 38% of the reports. The chronology of the facts was described in 88% of cases, and the search for the causes was present in 84%. However, this cause analysis was often incomplete, not exploring all the categories of causes. The most frequently explored domain was organisation (70% of the analyses) and patient-related causes were poorly explored (33% of the analyses). Overall, only 21 RCA reports (23%) included a complete search for the seven latent factor domains defined by the method.

**Table 3:** Characteristics of analysis reports reported to committee (*n* = 91).

	All EFCs		Per EFC	
	*n*	%	Median	[IQR]
Presentation of the analysis report	91	100	5	[1; 7]
Presentation format				
Orion format	72	79	5	[3; 6]
Oral	10	11	1	[1; 2]
Oral with visual support	8	9	4	[1; 7]
Methods of collecting data	35	38	1	[0; 4]
Individual interviews	32	35	4	[3; 4]
Debriefing	8	9	0	[0; 1]
Patient records	14	15	2	[0; 2]
Site visit	22	24	1	[1; 3]
Search for documents	13	14	1	[0; 1]
Chronology of the facts	80	88	5	[3; 7]
Description of the chronology	75	82	5	[2; 6]
Identifying errors	52	57	3	[1; 5]
Investigation of causes and contributing factors	76	84	6	[3; 6]
Organisation	64	70	5	[3; 6]
Working conditions	51	56	4	[2; 4]
Team functioning	50	55	3	[2; 5]
Policy	49	54	2	[1; 6]
Staff	49	54	3	[2; 5]
Guidelines, procedures	48	53	2	[1; 6]
Patients	30	33	2	[1; 3]
Other	9	10	0	[0; 1]
Proposals for corrective actions	86	95	6	[4; 7]

Abbreviation: IQR, interquartile range (i.e., 25^th^ and 75^th^ percentiles)

### Corrective actions

The annual number of corrective actions decided by each EFC ranged from 0 to 62, with a median of five (Table 4). The most frequent actions were to change an organisation (34%), write or modify a procedure (30%) or organise staff training (22%). The majority of actions had a designated person responsible (77%), who generally worked in the same department (70%). Only 26% of all corrective actions included a deadline for implementation.

**Table 4:** Characteristics of the actions adopted by the committee (*n* = 206).

	All EFCs		Per EFC	
	*n*	%	Median	[IQR]
Actions adopted by the EFC	**206**	100	5	[0; 13]
Action type:				
Organisation improvement	70	34	1	[0; 5]
Write or revise a procedure	61	30	1.5	[0; 3]
Train staff	45	22	1	[0; 2.5]
Improve a device	16	8	0	[0; 0.5]
Other	13	6	0	[0; 0]
Person in charge of the action:				
Member of department	145	70	1	[0; 8]
Other	15	7	0	[0; 1]
Undesignated	46	22	0.5	[0; 3]
Defined deadline	53	26	0	[0; 3.5]

Abbreviation: IQR, interquartile range (i.e., 25^th^ and 75^th^ percentiles)

### Factors related to the EFC productiveness

The most productive committees, defined as EFCs that decided at least five actions in the year, reviewed more events (109 versus 45, *p* = 0.02) and achieved more investigations (seven analysis reports versus one, *p*<0.001) (Table 5). The analysis reports provided root causes of the event more often (*p*<0.001). The most active committees designated a person in charge of the corrective action more often (*p*<0.001) and their actions had a deadline for implementation more often (*p* = 0.01).

**Table 5:** Comparison of characteristics of EFCs according to the number of actions decided in the year (< 5 versus ≥ 5).

	< 5 actions	≥ 5 actions	*P*
Department specialty; *n* (%)			0.07
Clinical department	9 (90)	5 (50)	
Medical-technical department	1 (10)	5 (50)	
EFC seniority, median [IQR], y	2 [1; 2]	1 [1; 2]	0.08
Number of attendees, median [IQR]	15 [9; 19]	20 [14; 22]	0.29
Number of events reported, median [IQR]	45 [33; 60]	109 [76; 142]	0.02
Number of analysis reports, median [IQR]	1 [0; 4]	7 [6; 8]	<0.001
Mode of presentation; *n* (%)			
Orion format	5 (50)	9 (90)	0.07
Other format	5 (50)	1 (10)	
Search for causes, median [IQR]	0 [0; 2]	6 [5; 7]	<0.001
Designated person in charge of action, median [IQR]	0 [0; 1]	8 [5; 18]	<0.001
Defined deadline, median [IQR]	0 [0; 0]	4 [0; 6]	0.01
Follow-up of previous actions, median [IQR]	3 [0; 7]	6 [2; 8]	0.14

Abbreviation: IQR, interquartile range (i.e., 25^th^ and 75^th^ percentiles); y, year

## Discussion

This study shows that nearly half of all medical departments voluntarily implemented an Experience Feedback Committee (EFC). Healthcare professionals adhere to the method that is implanted in a wide variety of medical departments. Reported adverse events are analysed and corrective actions are decided by the committees.

However, this picture is mitigated by the problems maintaining this activity in the healthcare teams’ routine. The number of meetings varied over time and from one department to another; some even had long periods of inactivity. Healthcare professionals explained these variations in activity by the departure of a leader who was not replaced and, above all, by the lack of time and resources. Indeed, carrying out investigations to identify the causes of events, as well as writing analysis reports, takes a lot of time for professionals who are already very busy.[14, 15]

The study also shows the sometimes significant deviations in practices compared to the theoretical functioning of the EFC. The EFC is based on a systems approach to patient safety and it provides a formal method for the root cause analysis (RCA) of adverse events. The Orion method, based on the Reason model, is close to the “Association of Litigation And Risk Management” (ALARM) method and includes the same steps.[16] Initiated in civil aviation, the Orion method was adapted to the field of healthcare by aviation safety experts. It is simpler than the ALARM method and, a priori, easier to use by healthcare professionals not specialised in risk management. However, the analysis of events did not always follow all the steps defined by the Orion method and the search for contributing factors was often incomplete and superficial. There was also a lack of planning the action selected by the committee and a failure to follow up the corrective actions decided previously.

This weakness found in the practice of RCA in the field of healthcare is reported by many authors.[14, 17–19] Earlier studies of RCA reports showed that this analysis often lacks depth and rigor, or that the method is rarely adequately applied.[17, 19] Overly simple or poorly designed action plans are insufficient to prevent the recurrence of incidents and may even generate new risks.[17, 20, 21] In addition, action plans are often not followed up, and when this monitoring exists, only part of the actions decided are effectively implemented.[14, 17]

This lack of rigor in RCA application might suggest that better training is needed to involve healthcare professionals more effectively in RCA within the team. More generally, these findings raise questions about the relevance of performing RCA by caregivers rather than by risk management experts.

When interviewing healthcare professionals who participated in RCA training programs, paradoxical responses were obtained.[14, 15, 19] On the one hand, these people express a very positive opinion of the method, which contributes to improving the safety of care and induces cultural changes.[14] On the other hand, the same people express difficulty in using RCA in practice. In addition to the lack of time and resources, healthcare professionals brought up difficulties involving interprofessional relations.[14, 15, 19] Indeed, the RCA interacts with a complex sociocultural context in which the investigation of a care-associated adverse event can be misunderstood.[17, 22] To preserve good interprofessional relations and avoid hierarchical tensions, the investigators remain on the surface of the issue and conceal certain profound sociopolitical and organisational problems.[17, 18, 22]

RCA effectively improves safety in various industries such as civil aviation. Because of this potential, RCA has become an important part of all healthcare safety management programs around the world.[15, 17] There is, however, no scientific evidence that RCA improves the safety of care.[15, 23] Studies that measured the impact of incident reporting systems or RCA use did not show any effect or only anecdotal effects.[23, 24] For example, Percarpio et al. studied 139 Veterans Affairs Medical Centers and found an association between RCA practice and the level of some safety indicators for postoperative complications,[25] but the study design could not assert that the relationship was causal. RCA’s limitations are also illustrated by examples of incidents that occurred after an identical incident was analysed and an action plan decided within the same department.[17]

In agreement with the literature, we found that the practice of RCA in the EFC is imperfect and that it would be illusory to expect short-term effects on the incidence of adverse events and patient safety. However, we hope that the EFCs will have long-term effects by increasing safety culture and learning by error. Indeed, the EFC is a particular mode of implementation of RCA in the field of healthcare that aims to directly involve members of the staff in the management of adverse events affecting their department. A person who reports an incident is invited to participate in the analysis and solution development and can then see the implementation of corrective actions and observe their effects. In this context, the professionals receive feedback on the reports, within a short feedback loop. This can help reduce a traditional barrier of incident reporting that is related to the opacity of reporting systems and the lack of feedback to the reporters about actions decided following such reports.[26] We support the hypothesis that the direct involvement of healthcare professionals in a learning-by-error system can be a strategy for the acquisition of values and behaviours that make up the safety culture.[26] In another study, we analyzed the association between patient safety culture, as measured by the Hospital Survey on Patient Safety Culture (HSOPS), and the care provider involvement in EFC activities.[9] We showed that EFC participants had a more highly developed patient safety culture, with nine of the 12 HSOPS dimension scores significantly higher than EFC non-participants. The three largest differences in the HSOPS score were related to the “feedback and communication about error”, organisational learning” and “non-punitive response to error” dimensions.[9]

We also hypothesise that the system-based approach to managing adverse events within a team is likely to improve the perception of the collective dimension of healthcare and thus foster teamwork. The social and cultural functions of the EFCs can be compared to those of the mortality and morbidity conferences when they are used to improve the quality and safety of care.[27, 28] Like the EFCs, the mortality and morbidity conferences can lead to the implementation of improvement action plans.[29, 30] Their contribution to fostering teamwork and enhancing the safety culture of healthcare professionals has been acknowledged.[27, 28, 31, 32]

The main limitation of this study is that it concerns only one hospital. It is probably not representative of the functioning of all EFCs in all French hospitals. However, this study is exploratory, it is the first one that analyses the functioning of several EFCs implanted in different medical specialties. It shows the difficulties of running a risk management system on a regular basis and opens up leads for carrying out broader studies on several hospitals.

We can also discuss the relevance of the criterion based on the number of actions decided to estimate the effectiveness of EFCs. This is an intermediate criterion, indicative of EFC functioning. To evaluate EFCs’ ability to improve care safety, it would be necessary to verify the actual implementation of these actions and their effect on adverse event incidence.

Another limitation is related to the retrospective collection of data, including missing data due to absent or incomplete reports. However, this lack of traceability is itself a result confirming how difficult it is for teams to follow a rigorous method.

In spite of these limitations, this study provides leads that may improve the functioning of EFCs. Although the method seems simple, its implementation requires training and it will be necessary to strengthen the training of healthcare professionals and to offer long-term methodological support by risk management specialists.

## Conclusion

The EFC is a way to involve healthcare professionals in system-based analysis of adverse events associated with medical care. The study identifies the limitations of this type of activity, which requires time for professionals and skills that are not easy to acquire. But the main barriers to implementing RCA in healthcare teams are psychological, social and cultural. However, we observe the approval of professionals persisting over time, and we remain hopeful that RCA will contribute to improving the safety culture of healthcare professionals.

## Supporting information

S1 Dataset (XLS)
